# Acceleration of Hyperspectral Skin Cancer Image Classification through Parallel Machine-Learning Methods

**DOI:** 10.3390/s24051399

**Published:** 2024-02-21

**Authors:** Bernardo Petracchi, Emanuele Torti, Elisa Marenzi, Francesco Leporati

**Affiliations:** Department of Electrical, Computer and Biomedical Engineering, University of Pavia, I-27100 Pavia, Italy; bernardo.petracchi01@universitadipavia.it (B.P.); emanuele.torti@unipv.it (E.T.); elisa.marenzi@unipv.it (E.M.)

**Keywords:** hyperspectral imaging, machine learning, support vector machine, random forest, eXtreme gradient boosting, GPU

## Abstract

Hyperspectral imaging (HSI) has become a very compelling technique in different scientific areas; indeed, many researchers use it in the fields of remote sensing, agriculture, forensics, and medicine. In the latter, HSI plays a crucial role as a diagnostic support and for surgery guidance. However, the computational effort in elaborating hyperspectral data is not trivial. Furthermore, the demand for detecting diseases in a short time is undeniable. In this paper, we take up this challenge by parallelizing three machine-learning methods among those that are the most intensively used: Support Vector Machine (SVM), Random Forest (RF), and eXtreme Gradient Boosting (XGB) algorithms using the Compute Unified Device Architecture (CUDA) to accelerate the classification of hyperspectral skin cancer images. They all showed a good performance in HS image classification, in particular when the size of the dataset is limited, as demonstrated in the literature. We illustrate the parallelization techniques adopted for each approach, highlighting the suitability of Graphical Processing Units (GPUs) to this aim. Experimental results show that parallel SVM and XGB algorithms significantly improve the classification times in comparison with their serial counterparts.

## 1. Introduction

Skin cancer represents one of the most predominant tumors [1], and in recent years, its occurrence has progressively increased. Such lesions are typically categorized into two main groups: melanoma skin cancer (MSC) and non-melanoma skin cancer (NMSC) [2]. Typically, this cancer type involves three types of cells: squamous, basal, or melanocytic cells.

MSC originates from melanocytes, cells located in the epidermis and responsible for skin color, thanks to melanin production. MSC can be further subdivided into three subtypes: superficial extension, lentigo maligna, and nodular tumor [3]. This is the rarest type of skin cancer, with, if not promptly detected, the highest growth speed and, consequently, is very difficult to treat [4]. Therefore, doctors and surgeons need fast, reliable diagnostic systems for this kind of pathology.

The traditional diagnosis procedure is biopsy, which consists in the removal of a sample of tissue from the living body, followed by histopathological inspection [5,6], representing an onerous and time-consuming process [5,6,7].

To face these problems, minimally intrusive techniques have been investigated, including hyperspectral imaging (HSI), acquiring information about a scene both in the spatial and in the spectral domain [8]. In fact, a hyperspectral image is represented by a so-called hypercube containing the spectral information of every pixel over a specific wavelength range. HSI allows precise material identification [9] by measuring the fraction of the incident electromagnetic radiation reflected by the surface (reflectance). This is due to the characteristic variation in the reflectance over the wavelength typical of each material, which is called the spectral signature [10]. In contrast with traditional imaging techniques, HSI allows the acquisition of images with a large number of spectral bands both within the visible and non-visible range. This means that the acquired images contain much more information compared to traditional ones, such as RGB images, and can lead to better performances [11].

However, although the development of accurate tools in the medical field is fundamental, timing requirements should also be taken into consideration when providing a quick diagnosis is necessary. Indeed, the prompt detection of skin lesions facilitates their treatment and increases the probability of survival of the patients.

To achieve this goal, many researchers [12,13,14,15,16,17] have exploited different kinds of devices suitable for parallel elaboration and computation when the data size is high. Among these, Graphical Processing Units (GPUs), used in different scientific applications [18,19], represent a suitable technology in the field of medical image processing. In addition, compared with other devices such as Field Programmable Gate Arrays (FPGAs), GPUs usually offer a bigger parallel factor due to their high memory bandwidth [20].

Existing works in the literature have focused on the classification of HSI skin cancer images by adopting machine-learning (ML) and deep-learning (DL) methods [11,16,21,22,23,24,25,26,27,28,29,30,31].

In [16], a classification chain based on K-means, Spectral Angle Mapper (SAM), and SVM was considered. The authors also implemented several parallel versions of their classification system exploiting multicore and many-core technologies.

The research in [31] implemented SVM, RF, and XGB, obtaining a mean classification accuracy of 97%, considering only the model’s optimization and not the algorithms’ parallelization.

Several DL models have been adopted in [32], namely, ResNet-18, ResNet-50, ResNet-101, a ResNet-50 variant, U-Net, and U-Net++ architectures. Since neural networks are time-consuming and computationally expensive, a parallel version of the U-Net++, resulting in the best predictive approach, has been implemented using a low-power NVIDIA Jetson GPU. This parallel version has achieved adequate classification performance satisfying real-time constraints with a low power consumption.

Some works related to ML method parallelization can be found in [16,33], where parallel versions of SVM and XGB have been developed for HSI image classification.

In this paper, we propose the optimization and parallelization of three popular ML methods to accelerate the HSI skin cancer image classification using the Compute Unified Device Architecture (CUDA), a framework for parallel elaboration developed by NVIDIA. More specifically, the considered approaches are SVM, RF, and XGB, which offer a good performance in classifying HSI images when the dimensions of the dataset are limited [31,34]. Furthermore, the works in [16,33,35] showed a great reduction in the classification time developing parallel versions of SVM and XGB, even achieving real-time processing.

This work presents the parallelization techniques implemented on different NVIDIA GPU devices including a GeForce RTX 2080 GPU, a GeForce RTX 4090 GPU, and a cluster composed of five nodes of three Tesla A16 GPUs. Performance differences between the devices in the classification of HSI skin cancer images have also been highlighted. Indeed, GeForce RTX 2080 and 4090 GPUs are optimized for graphics applications, while the cluster is designed for scientific calculations. In particular, the GeForce RTX 4090 is characterized by the latest-generation architecture (Ada Lovelace), while the GeForce RTX 2080 features an older architecture (Turing) and is cheaper than the previous one. Lastly, each Tesla A16 features an Ampere architecture.

Experimental results show a significant improvement of the parallel version of SVM and XGB compared to their serial counterparts, with a speed-up of 130x and 1.4x, respectively, confirming that GPUs represent a valid technology in accelerating the medical diagnosis process.

This manuscript is organized as follows. Section 2 describes the HSI skin cancer dataset and the adopted ML algorithms. Furthermore, the adopted techniques to perform the serial and the parallel inference of the algorithms, and the architectures of the adopted devices are shown. The obtained results are illustrated in Section 3, while Section 4 presents the discussions, and Section 5 provides conclusions and future developments.

The main contributions of this paper are the following: description of the parallelization of the SVM, RF, and XGB methods targeting GPUs; parallelization on different devices, considering the most recent architectures developed by NVIDIA; and comparison of the results with the state of the art, highlighting the improvement of skin cancer diagnosis through parallel image processing.

## 2. Materials and Methods

### 2.1. Hyperspectral Sensors and the Skin Cancer Dataset 

The evolution of hyperspectral sensors has resulted in the creation of various platforms, specialized for particular applications and operational needs. The four main sensor types, namely pushbroom, whiskbroom, stereoscopic, and snapshot are fundamental to the hyperspectral imaging landscape [36,37,38]. Pushbroom sensors function through constant scanning of the scene using a linear or 2D array of detectors. As the platform moves, the sensor captures spectral information for every pixel in the scene, resulting in a continuous spectral image. This technique enhances both spatial and spectral resolution, making pushbroom sensors highly suitable for applications that demand a thorough analysis of specific regions [39].

Whiskbroom sensors operate similarly to pushbroom ones, except for their scanning mechanism. Rather than recording an entire line at once, whiskbroom sensors collect data one point at a time. The sensor sweeps across the scene, gathering spectral information for each point sequentially. Whiskbroom sensors are celebrated for their adaptability and are frequently utilized in airborne and spaceborne reconnaissance [40].

Stereoscopic hyperspectral sensors employ several detectors to capture images from marginally divergent viewpoints. By leveraging stereoscopic vision, these sensors provide not only spectral data but also depth information. This facilitates the creation of 3D models and improves the interpretation of intricate surroundings, such as hilly terrains or urban landscapes [41].

Snapshot sensors, also referred to as snapshot hyperspectral imaging systems, obtain a complete spectral image with a single exposure. This is accomplished through cutting-edge optical designs that record data concurrently for all spectral ranges. Snapshot sensors enable quick data acquisition and are ideal for dynamic scenarios or situations needing promptly available spectral information [42].

A thorough knowledge of the peculiar characteristics of each hyperspectral sensor is crucial to select the most appropriate technology for a particular application. Concerning skin cancer detection, the snapshot sensor is the best choice since it acquires the whole images in a single exposure [25,36].

The HSI skin cancer dataset used is the one considered in [16,21,31,43]; it contains 76 images of skin lesions from 61 subjects, 40 of which are benign and 36 are malignant. They were acquired with a snapshot camera (Cubert UHD, Cubert GmbH, Ulm, Germany) able to cover the 450–950 nm range, distributed over 125 spectral channels [30]. The images were collected in two hospitals of the Canary Islands, Spain: the Hospital Universitario de Gran Canaria Doctor Negrín and the Complejo Hospitalario Universitario Insular-Materno Infantil. The image labelling was led by experts such as dermatologists and pathologists according to the taxonomy described in [32].

The spectral signatures among different patients have been normalized as illustrated in [32] to mitigate the variations in illumination conditions. At the end of preprocessing, the spectral signatures contain 116 bands with values in the range [0, 1].

**Figure 1:**
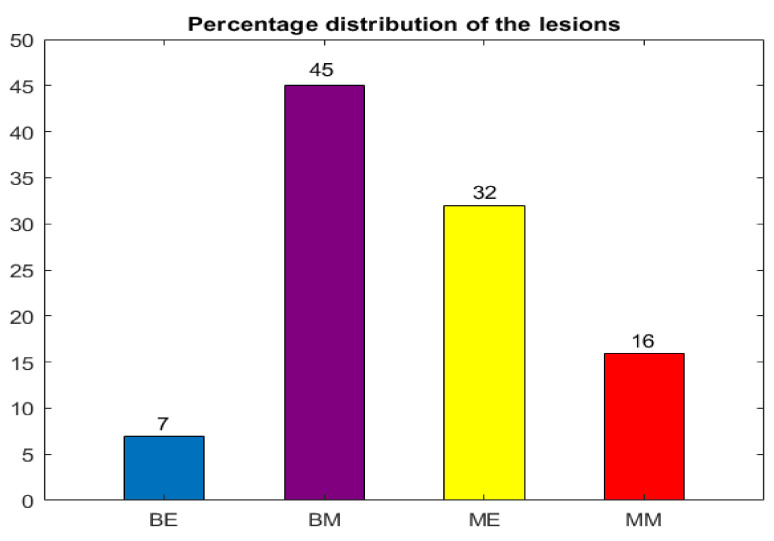
Percentage distribution of each lesion.

Figure 1 shows the percentage distributions of the skin lesions that include four possible classes: Benign Epithelial (BE), Benign Melanocytic (BM), Malignant Epithelial (ME), and Malignant Melanocytic (MM).

**Figure 2:**
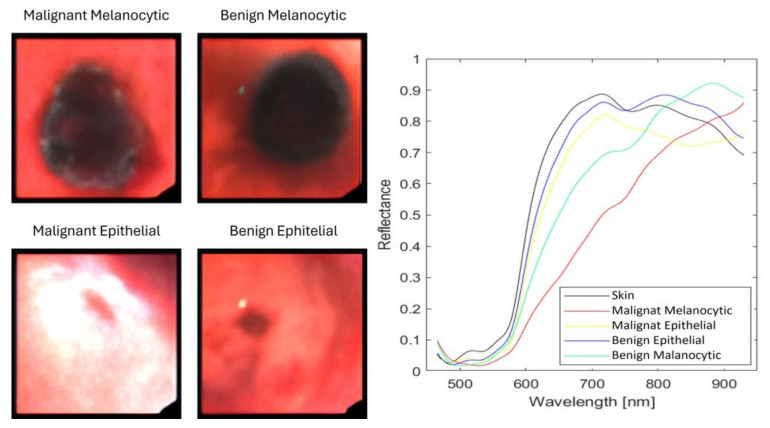
Synthetic RGB images taken from the database to represent each lesion and the mean spectra of the pixels.

Figure 2 shows four images taken from the dataset representing one of the considered lesions, together with the mean spectral signatures of the hyperspectral pixels.

### 2.2. Machine-Learning Methods

This section gives a general overview of the SVM, RF, and XGB methods adopted to classify the HSI skin cancer images. Specifically, theoretical aspects of the three algorithms will be presented.

#### 2.2.1. Support Vector Machine

SVM is a supervised machine-learning method proposed by Vapnik and extensively used for classification and regression tasks [44,45,46]. Originally, SVM performs binary classifications and aims to find the hyperplane which splits the dataset into discrete classes according to the given training samples [46]. The data points with the minimum distance from the hyperplane are called support vectors (SVs). For multiclass classification, SVM breaks down the multiclass problem into multiple binary classification ones, solving the following equation:

$$\underset{w,b,\zeta }{\min}\frac{1}{2}w^{T}w+C\sum_{i=1}^{n}\zeta_{i}$$

(1) $$subject\;to\;y_{i}\left(w^{T}x_{i}+b\right)\geq 1-\zeta_{i},$$

$$\zeta_{i}\geq 0\;with\;i=1,\ldots ,n$$

where w is the support vectors, C is the penalty term, $\zeta_{i}$ is the distance error from the correct margin, y is the classes, b is the margin, $x_{i}$ is the training vectors, and n is the number of training samples. Intuitively, the goal is to maximize the margin by minimizing $w^{T}w$, while incurring a penalty when a sample is misclassified.

The minimization problem described by Equation (1) can be transformed into a dual problem given by Equation (2):

$$\underset{\alpha }{\min}\frac{1}{2}\alpha^{T}Q\alpha -e^{T}\alpha$$

(2) $$subject\;to\;y^{T}\alpha =0,$$

$$0\leq \alpha_{i}\leq C\;with\;i=1,\ldots ,n$$

where e is a vector of all ones, and Q is an n by n positive semidefinite matrix whose elements are defined in Equation (3):

(3) $$Q_{ij}=y_{i}y_{j}K(x_{i}x_{j})$$

K is the kernel function that maps the data from a low-dimensional space to another space with high dimensions. Once the optimization problem is solved, the output of decision function for a given sample x becomes:

(4) $$\sum_{i€SV}\alpha_{i}K\left(w_{i},x\right)+b$$

where $\alpha_{i}$ is the dual coefficients. The sign of Equation (4) gives the binary classification, while the multiclass classification is achieved according to the “one-vs.-one” strategy by repeatedly applying Equation (4).

#### 2.2.2. Random Forest

RF was first introduced by Leo Breiman [47]. It is a popular ensemble learning algorithm used for both classification and regression tasks. It combines the predictions of multiple decision trees to improve the predictive accuracy and control over-fitting. Specifically, each tree performs a “partial” prediction, and the class with the most votes becomes the final prediction. Using a random subset of data and features, each decision tree in the RF is built recursively by splitting the data according to various criteria (e.g., Gini impurity or information gain) until a stopping criterion is met. The latter can be a maximum tree depth, a minimum number of samples required to split a node, or a minimum number of samples required in a leaf node.

#### 2.2.3. eXtreme Gradient Boosting

XGB is an ensemble learning algorithm similar to RF. It is based on a generalized gradient boosting method, and is used for classification, regression, and ranking tasks [48,49,50]. It provides highly accurate classifications by combining the predictions of multiple weak predictive models, typically decision trees. One of the strong points of XGB is the sequential addition of new models correcting the mistakes made by previous models. Particularly, it optimizes a specific loss function by computing its gradient compared to the predicted values. XGB builds N trees per class; the outputs of the trees belonging to the same class are summed. The soft-max function is then applied to the outputs to obtain the probability values of the class. The class with the biggest value is the final prediction.

### 2.3. CPU and GPU Technologies

This section describes the architectures and the main features of the CPU and GPU devices employed for the inference implementation of the three algorithms. For the serial inference, we used an Intel Core i9-13900K with a clock frequency of 3 GHz. It is based on the Raptor Lake architecture developed adopting an Intel 7 processor (10 nm), with 24 cores, 32 threads, and 32 MB and 36 MB of L2 and L3 cache memory, respectively. The maximum bandwidth achievable is 89.6 GB/s.

The first two GPU devices considered for the parallel inference were an NVIDIA GeForce RTX 2080 and an NVIDIA GeForce RTX 4090, optimized for graphics applications.

The NVIDIA GeForce RTX 2080 is based on the Turing architecture with 2944 CUDA cores and a clock frequency of 1.5 GHz. Other components of this device include 184 texture units, 64 Render Output Units (ROPs), 368 tensor cores, 46 ray tracing (RT) cores, and 8 GB of GDDR6 modules. The maximum bandwidth achievable is 448 GB/s.

The NVIDIA GeForce RTX 4090 is supported by the Ada Lovelace architecture with 16,384 CUDA cores and a clock frequency of 2.2 GHz. It also contains 512 tensor cores, 176 ROPs, and 128 RT cores. The memory dimension is 24 GB (GDDR6X), and the maximum bandwidth is 1008 GB/s.

The last GPU device considered is a cluster dedicated to the scientific calculation composed of five nodes of three NVIDIA Tesla A16s. Each GPU of the cluster is equipped with four chips and features the Ampere architecture. Every chip of the GPU has 1280 CUDA cores, 40 tensor cores, 16 GB of GDDR6, and a memory bandwidth of 200 GB/s.

### 2.4. CPU Inference

The inference of the algorithms described in Section 2.2 has been implemented using the best parameters obtained after the training phase as detailed in [31]. Visual Studio 2022 Integrated Development Environment (IDE) was used, adopting the C language.

The serial implementation has been used as a basis for the parallel inference described in Section 2.5.

#### 2.4.1. SVM Inference

The SVM inference consisted in the implementation of Equation (4). The dual coefficients, the margin, the support vectors, and the type of kernel function have been identified after both the training and the parameters tuning described in [31]. The Radial Basis Function (RBF) resulted as the most appropriate kernel function, and it is represented by the following equation:

(5) $$K\left(w_{i},x\right)=e^{-\gamma {\left||w_{i}-x|\right|}^{2}}$$

where γ is the kernel parameter, whose best value obtained after the training was 10.

The steps executed to perform the SVM inference can be summarized as follows:

1. Kernel calculation for the sample to classify according to Equation (5);
2. Multiplication between the obtained kernel and the dual coefficients adding the bias b;
3. Pixel classification through the “one-vs.-one” strategy.

The pseudo-code of the SVM inference is reported in Algorithm 1. Lines 2 to 4 perform the kernel calculation by evaluating the squared Euclidean distance between the support vectors and the sample to classify. The second step is executed in lines 6 to 10, where the distance of the sample from the hyperplane is calculated according to Equation (4). Due to the nested loops, the distance is calculated $n_{class}\;*\;(n_{class}-1)/2$ times. With $n_{class}=5$, 10 values of the distance are obtained. Lines 12 to 21 show the last step that aims to perform the final prediction by observing the sign of the 10 values of the distance: if $d_{ij}$ is positive (negative), then class i wins (loses) over class j, and the array ${score}_{i}$ (${score}_{j})$ is incremented by one. Finally, line 21 finds the index of the maximum value in the array ${score}_{i}$, or rather, the class obtaining the greatest number of scores.
**Algorithm 1** Serial implementation of Support Vector Machine**Input**: γ → Kernel parameter ${DC}_{ij}$ → Dual coefficients matrix $w_{i}$ → Support vectors matrix x → Pixel to classify b → Bias 1: Step 1:Kernel calculation 2: $for\;i=0\;to\;n_{sv}-1$ 3:       $K\left(w_{i},x\right)=\exp\left(-\gamma *{\left\|w_{i}-x\right\|}^{2}\right);$ 4: **end** 5: Step 2:Distance of the sample from the hyperplane 6: $for\;i=0\;to\;n_{class}-1$ 7:        $for\;j=i+1\;to\;n_{class}-1$ 8:          $d_{ij}=\sum_{i€SV}DC_{ij}*K(w_{i},x)+b;$ 9:       **end** 10: **end** 11: *Step 3: “One vs. one” strategy* 12: $for\;i=0\;to\;n_{class}-1$ 13:       ${score}_{i}=0$ 14:       $\;for\;j=i+1\;to\;n_{class}-1$ 15:             $ifd_{ij}>0$ 16:                    ${score}_{i}++;$ 17:             else 18:                   ${score}_{j}++;$ 19:       **end** 20: **end** 21:  $Find\;imax,\;index\;of\;the\;{score}_{i}\;maximum$ **Output**: imax


#### 2.4.2. RF Inference

The core of serial RF inference is a recursive function representing the tree structure. According to the obtained trained values of the features, the thresholds, as well as the left and right children’s nodes of each parent node, the execution follows a specific path in the tree. If the execution ends in a non-leaf node, the function is repeated and drives the execution to the next node depending on the left and right children’s values. The recursion stops when the execution ends in a leaf containing the output. The output of this function is an array of 5 elements containing the probability values of the pixel of belonging to each class. Then, a second function was realized with the goal to execute the tree structure N times, where N is the number of decision trees. Therefore, each tree makes its prediction on the pixel, and the class having the greatest number of votes is the final prediction. The number of decision trees used in this work is 425, obtained after the training phase. The pseudo-code of RF inference is shown in Algorithm 2. Line 2 corresponds to the tree_structure function that outputs the probability array (prob_array) exploiting the features, thresholds, and left and right children’s node (input_data). Lines 4 to 8 perform the forest in which, at each iteration, the tree_structure function runs and the index of prob_array maximum is obtained. At the end of the iterations, the array class contains the number of votes per each class. The final prediction is the most voted class and is obtained in line 9.
**Algorithm 2** Serial implementation of Random Forest**Input**: input_data → Features, thresholds, left and right            children’s nodes 1: *Step 1: Development of the*
tree_structure function 2: The single tree outputs prob_array 3: *Step 2: Building of the forest* 4: $for\;i=0\;to\;n_{trees}-1$ 5:  tree_structure(input_data,prob_array,i); 6:   Find max, index of prob_array maximum 7:   ${class}_{max}++;$ 8: end 9: Find imax, index of the class maximum **Output**: imax


#### 2.4.3. XGB Inference

XGB is based on the same tree_structure function of the RF, but in this case, the output is a single value. The forest structure function builds N decision trees for each class; each tree improves the output of the previous tree (belonging to the same class) by considering its prediction mistakes. The optimal number of decision trees obtained after the training was 400, so the forest structure function builds 2000 decision trees overall.

The outputs of the decision trees belonging to the same class are summed. In Algorithm 3, the pseudo-code of the XGB inference is shown. Line 2 is related to the tree_structure function that outputs the probability value of the single tree. Then, the forest function is described in lines 4 to 8, where the sums of the outputs of the trees belonging to the same class are stored in the $Z_{i}$ array of 5 elements. Lines 10 to 18 determine the final probability array $P_{i}$ according to the soft-max function reported in Equation (6). The index of $P_{i}$ maximum is the final prediction according to line 19.

(6) $$P[i]=\frac{ZE[i]}{\sum_{j=0}^{n_{class}}ZE[j]}$$

**Algorithm 3** Serial implementation of eXtreme Gradient Boosting**Input**: input_data → Features, thresholds, left and right            children’s nodes 1: *Step 1: Development of the*
tree_structure function 2: The single tree outputs the probability value of its class 3: *Step 2: Building of the forest* 4: $for\;i=0\;to\;n_{class}-1$ 5:  $for\;e=0\;to\;n_{trees}-1$ 6:     $Z_{i}+=tree\_strucutre(input\_data,\;e*\;\;n_{class}+i)$; 7:  end 8: end 9: Step 3:Final probability array through soft−max⁡function 10: $for\;i=0\;to\;n_{class}-1$ 11:    ${ZE}_{i}=\exp\left(Z_{i}\right);$ 12: **end** 13: $for\;i=0\;to\;n_{class}-1$ 14:    $z=\sum_{i€n_{class}}{ZE}_{i};$ 15: **end** 16: $for\;i=0\;to\;n_{class}-1$ 17:    $P_{i}={ZE}_{i}/z;$ 18: **end** 19: $Find\;imax,\;index\;of\;the\;P_{i}\;maximum$ **Output**: imax


### 2.5. GPU Inference

This section describes the parallel inference for the SVM, RF, and XGB algorithms. We adopted the GPU devices described in Section 2.3 and Visual Studio 2022 with CUDA C language.

In the following sections, we will explain some essential terms to define the basic components of the CUDA language. First, we must define the kernel (a CUDA function) that, when called, is executed in parallel by N different CUDA threads. Another important component is the thread block containing a group of threads executed concurrently. The threads belonging to the same block can cooperate through synchronization barriers. A thread block uses the shared memory for inter-thread communication and the data sharing. Finally, a grid is an array of thread blocks executing the same kernel; it reads and writes in the global memory of the GPU. Each thread and block can be identified through the *threadIdx = (threadIdx.x*, *threadIdx.y*, *threadIdx.z)* and *blockIdx = (blockIdx.x*, *blockIdx.y*, *blockIdx.z)* coordinates, respectively. The dimension of the thread block is defined by the *blockDim = (blockDim.x*, *blockDim.y*, *blockDim.z)* array.

#### 2.5.1. Parallel SVM

The most computationally expensive operations in SVM are *Step 1* and *Step 2* of Algorithm 1 in Section 2.4.1. *Step 1* involves the SV matrix (116 × 47,220) and the image to classify (2500 × 116), while *Step 2* performs the product between the obtained kernel (2500 × 47,220) and the dual coefficients matrix (47,220 × 4).

*Step 2* was performed through a CUDA kernel using a number of blocks equal to $(N+n_{threads}-1$)/$n_{threads}$ with $n_{threads}=32$ and N being the number of SVs. The choice to use 32 as the number of threads is because the basic unit of execution in an NVIDIA GPU is the warp, a collection of 32 threads executed simultaneously by a Streaming Multiprocessor (SM) of the GPU. Therefore, the resulting number of blocks was 1476. The pseudo-code of Algorithm 4 below represents the kernel calculation through the CUDA syntax.
**Algorithm 4** Kernel calculation**Input**: γ → Kernel parameter $w_{i}$ → Support vector matrix x → Pixel to classify 1: i
= blockIdx.x * blockDim.x + threadIdx.x 2: if i < $n_{sv}$ 3:
   $for\;i=0\;to\;n_{bands}-1$ 4:     ${d_{i}=\left\|w_{i}-x\right\|}^{2}$ 5:   **end** 6: $K\left(w_{i},x\right)=exp(-\gamma *d_{i})$ **Output**: $K\left(w_{i},x\right)$


In line 1, the variables *blockIdx.x* and *threadIdx.x* indicate the current block and thread identifier, while *blockDim.x* is the block dimension along the *x*-axis as described in Section 2.5. In line 4, the squared Euclidean distance $d_{i}$ is shown; each thread performs the difference between an element of the SV matrix $w_{i}$ and an element of the sample to classify x in parallel. Finally, in line 6, the kernel $K\left(w_{i},x\right)$ is obtained.

Then, *Step 2* was implemented by adopting the *cublasSgemm* and the *cublasSaxpy* functions (from the cuBLAS library) explicitly designed for matrix operations: the first has been used to perform the multiplication between the kernel and the dual coefficients matrix, the second to sum the obtained result and b. The result of this step was a vector of 10 elements containing the outputs of the decision function (see Equation (4)). *Step 3* was performed employing 1 block of 5 threads (1 per class), whose task was to apply the “one-vs.-one” strategy. Finally, the *cublasIsamax* function has been used to determine the final prediction.

#### 2.5.2. Parallel RF

For the parallel version of RF, the intrinsic nature of decision trees that is based on sequences of *if–else* statements causes threads divergence, representing a challenge that did not allow the parallelization of the tree_structure function. Therefore, such function has been declared as a device function using the CUDA keyword __device__, meaning that the function is called by the GPU.

The forest structure was realized with a CUDA kernel composed of 425 blocks of 1 thread, with one block for each decision tree and every block having only one thread in order to avoid the potential thread divergence in the tree_structure function.

The pseudo-code in Algorithm 5 represents the parallel RF inference. Line 2 refers to the serial RF tree_structure with the addition of the __device__ declaration, as mentioned above. Lines 4 to 6 perform the forest where each block builds a decision tree and outputs the prediction (max) for that same tree. Furthermore, to prevent race conditions in filling the class array, line 6 performs the *atomicAdd* operation to add the value 1 to all the elements of the array. In line 7, the final prediction imax is obtained through the *cublasIsamax* function.

**Figure 3:**
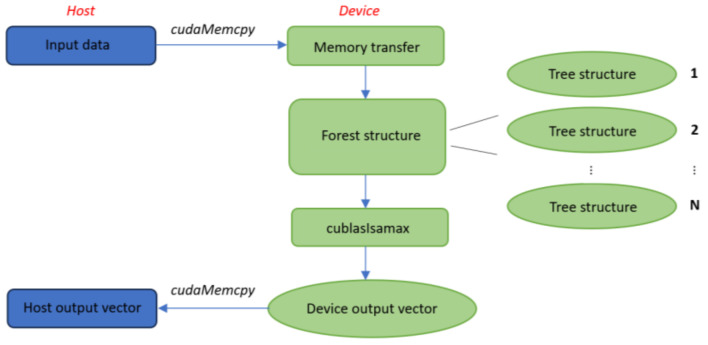
Flow diagram of parallel RF classifier.

Figure 3 shows the flow diagram of the RF classifier and how it is divided between host and device. The input data, stored in the host, are transferred in the device memory through the ***cudaMemcpy*** function, thus representing the input to the forest structure device function, where each block implements a decision tree by calling the tree_structure function. After that, the ***cublasIsamax*** function has been used to make the prediction for each specific pixel. Since the device output vector contains the predictions of every pixel of the image, its dimension is 2500. At last, the device output vector is transferred to the host memory.

#### 2.5.3. Parallel XGB

To perform the parallelized version of the XGB, the forest structure function has been designed similarly to the parallelized RF: 2000 blocks have been adopted, each including 1 thread, and launching the tree structure function. The values obtained for each block have been stored in the vector Z. Then, the reduction technique has been used to sum the elements of Z related to the same class. To perform this task, the “sequential addressing” strategy has been implemented. The code below shows the sequential addressing reduction technique.

In Code 1, for each class, 400 elements (*n_estimators)* of Z are transferred to the GPU shared memory through the array S. Then, the ***for*** loop reduces the entire upper portion of the array S to the entire lower portion of S. With 512 values, the upper 256 values are reduced into the lower 256 values. Then, the upper 128 values of the lower 256 values from before are reduced with the lower 128 values. The loop ends when the sum of all the elements of the array is obtained and stored in the first element of S.
**Code 1** Sequential Addressing Reduction**Input**: tid, e, b  → indexes of the threads and blocks ncl → number of classes 1: int tid=threadIdx.x; 2: __shared__ float S[512]; 3: int e=blockIdx.x ∗ blockDim.x+threadIdx.x; 4: int b=blockIdx.y; 5: if (tid < ***n_estimators***) 6:  S[tid]=Z[e ∗ ncl+b]; 7: __syncthreads();
8: for (s=blockDim.x/2;s>0;s≫=1){ 9:    if (tid < s) 10:  S[tid]+=S[tid+s]; 11:  __syncthreads(); 12:} **Output**: S


The reduction was executed using a 2D grid composed of 1 block of 512 (512 being the first power of 2 greater than 400) threads for the *x*-axis, and 5 blocks of 1 thread for the *y*-axis. Each thread of the *x*-axis transfers one element of Z to the shared memory and sums two elements of Z, while the 5 blocks of the *y*-axis iterate over the classes. Algorithms 4 and 5, related to SVM and RF, respectively, involve a single index in performing their kernels; therefore, the use of a 1D grid was considered sufficient. In the reduction process, XGB involves two independent indexes, e and b, related to the elements of the S array and to the classes, respectively; as a consequence, a 2D grid has been identified as more suitable compared to a 1D grid.
**Algorithm 5** Parallel Random Forest**Input**: input_data → Features, thresholds, left and right             children’s nodes 1: *Step 1: Development of the*
device tree_structure function 2: The single tree outputs max, the prob_array maximum index 3: *Step 2: Building of the forest* 4: i=blockIdx.x; 5: max=tree_structure(input_data, prob_array, i); 6: $atomicAdd\left(\&{class}_{max},\;1.0\right);$ 7: Find imax, index of the class maximum **Output**: imax


**Figure 4:**
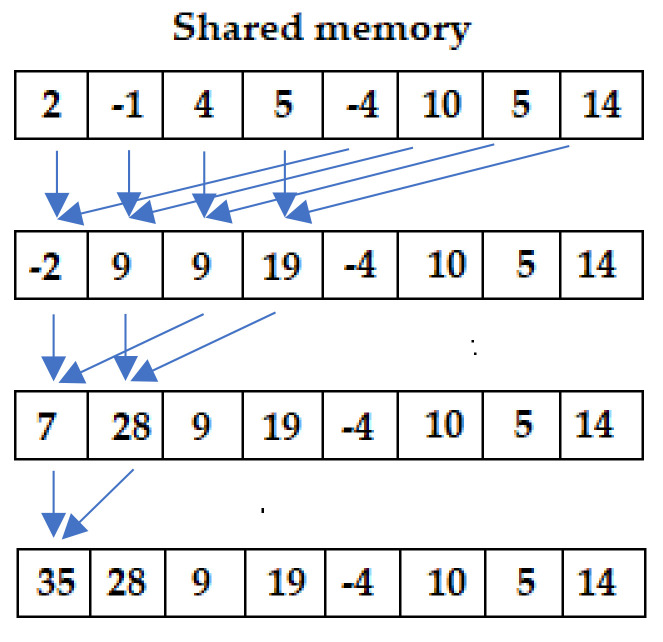
Example of sequential addressing reduction technique.

The sequential addressing approach solves the warp’s divergence and shared memory bank conflict problems of the interleaved addressing reduction. Figure 4 exemplifies the concept of sequential addressing reduction.

To conclude, the final probability array P of Equation (6) was obtained using a CUDA kernel composed by 5 blocks of 1 thread.

## 3. Results

The inference part of SVM, RF, and XGB methods has been implemented in a serial and a parallelized version using C and CUDA languages, respectively. The programs have been developed with the Microsoft Visual Studio 2022 IDE and the CUDA 11.7 toolkit for the NVIDIA GeForce RTX 2080 GPU and the CUDA 12.0 toolkit for the NVIDIA Tesla A16 and NVIDIA GeForce RTX 4090 GPUs. The serial version was compiled with the v143 compiler of Visual Studio, while the parallel code was compiled with the NVCC compiler included in the toolkit. The compiler configuration has been set to release mode, meaning that the optimizations are enabled, and that the full debugging information is not included. Furthermore, we have set the code generation option of the CUDA compiler to 7.5, 8.6, and 8.9 values corresponding to the compute capability of the NVIDIA GeForce RTX 2080, NVIDIA Tesla A16, and NVIDIA GeForce RTX 4090 GPUs. This option allowed us to fully exploit the architectures of the respective GPUs.

The SVM, RF, and XGB inference has been tested using 10 HSI skin cancer images, all having dimensions of 50 × 50 pixels and 116 bands; this dataset contains all the possible skin lesions.

Specifically, the average classification time of such images has been measured for each algorithm and for all the adopted technologies. All the average classification times with the standard deviations and the speed-up (in brackets) are reported in Table 1.

It is worth noting that the parallel SVM features the greatest speed-up. In fact, all GPU devices have obtained valid results for this algorithm: a speed-up of 32x, 11x, and 130x turned out for the GeForce RTX 2080, Tesla A16, and GeForce RTX 4090, respectively. This confirms that parallelizing SVM is an appropriate solution for the acceleration of skin lesions’ detection.

Parallel XGB has outperformed its serial counterpart when using both the GeForce RTX 2080 and GeForce RTX 4090 GPUs, achieving a speed-up of 1.19x for the first and 1.39x for the second device conversely. The cluster has not accelerated the serial version, its average execution time being 1.17 s, whereas 1.43 s is the average execution time of the parallelized version.

Finally, RF is the only algorithm that has not shown improvements; however, some observations should be made: the intrinsic nature of RF did not allow the tree structure to be parallelized since it is based on *if–else* sequences. Hence, this algorithm is not fully parallelizable. Moreover, the number of decision trees used in this work was 425, which is not as big as it should be to adequately exploit the benefits of parallel computing.

NVIDIA GeForce RTX 4090 GPU resulted as the most performant among the GPUs, due to its high number of CUDA cores (16,384) and to its latest-generation architecture, the Ada Lovelace.

As already said, the university cluster achieved the worst performance for all algorithms, probably because the code developed for the parallel inference has not exploited the full computational power of the cluster. Indeed, the cluster is composed of five nodes of three Tesla A16 GPUs, while our code employed the use of one out of four chips equipped on each single GPU.

## 4. Discussion

To compare the results of our methods with the state of the art, the works proposed in [16,33] can be considered. The authors of [16] have developed a hybrid classification system based on K-means, SAM, and SVM using the same dataset here described. In particular, they implemented several parallel versions of their system using an NVIDIA GeForce RTX 2080 GPU (the same employed in this work) and an NVIDIA Tesla K40 GPU. The best performance was achieved through the version performing the K-means in CUDA using the NVIDIA GeForce RTX 2080 GPU and the SVM in OpenMP. To evaluate the performance, the authors considered nine images and measured the classification times of each image as the mean of five executions. They reported a diagram showing that the classification times of their system were approximately 1 s. However, the SVM implementation in [16] had to classify only a limited number of pixels of the images; namely, the pixels clustered as pigmented skin lesions from the K-means stage. In contrast, this work’s SVM classified all the 2500 pixels of the images, discriminating between five different classes. Indeed, the computational complexity of the SVM adopted in [16] is lower than the one described in this work. Not only the number of elements to classify is lower, but also the hyperparameters are different, since a higher number of support vectors is needed by the SVM adopted in this paper.

In [33], a parallel XGB version was developed using an NVIDIA Quadro P4000 to classify the Pavia University (PU), GRSS-DFC2013 Houston (GH13), and GRSS-DFC2018 Houston (GH18) datasets. All three datasets are based on a single HSI image. The PU image features a dimension of 610 × 340 pixels and 103 channels, while the GH13 image is a cube of dimensions 349 × 1905 × 144. Finally, the GH18 Houston image has 4172 × 1202 pixels and 48 bands. The times taken to classify these images were 6.67 s, 31.05 s, and 347.30 s for the PU, GH13, and GH18 datasets, respectively. Given the big difference between the number of samples and features considered in the datasets of [33] and the one of this work, a quasi-linear relation between the images size and the processing times is observed. Indeed, the structure of XGB is poorly parallelizable, and the performances are strictly related to the number of features and trees. In the proposed work, since the data dimensionality is lower than that of [33], the number of features and trees is small. Moreover, as described in Section 2.5.3, the parallelization is based on assigning each tree to a block, whilst instead, [33] uses a standard approach.

To the best of the authors’ knowledge, no prior parallel version of RF has been developed in the HSI field.

Table 2 summarizes the prediction times of this work and the results obtained in the literature.

## 5. Conclusions

In this work, a serial and a parallel inference of the SVM, RF, and XGB algorithms to classify a dataset of HS skin cancer images have been proposed. The serial inference has been implemented employing the CPU Intel Core i9-13900K, and to accelerate the serial classification, three different GPUs have been employed: the NVIDIA GeForce RTX 2080, the NVIDIA Tesla A16, and the NVIDIA GeForce RTX 4090.

The results show that our work can significantly accelerate medical diagnosis through image processing techniques. In fact, the parallel versions of both SVM and XGB lead to an acceleration very significant in the case of the most complex SVM and minor but not neglectable in the case of the less challenging XGB. In any case, this experimentation confirms the validity of the approach used in [16] and in [38] even in case of a problem featuring a low parallelizable algorithm applied to a small dataset with a low number of trees. Again, it is possible to say that hyperspectral image processing can support doctors in timely detecting skin lesions, planning an opportune therapy, and helping surgeons during interventions.

Future works will focus on multi-GPU programming to exploit the full computational power of the cluster, since we only used one out of four GPUs of one NVIDIA Tesla A16. Furthermore, integrated GPU solutions will be explored, such as the NVIDIA Jetson, that is a System on Module (SoM) that features small dimensions, high performance, and embedded CPU, GPU, and memory in a single board. Lastly, datasets with a higher number of patients will be considered to better validate the proposed approach.

## Figures and Tables

**Table 1:** Average classification times for SVM, RF, and XGB for all the CPU and GPU devices.

	SVM [s]	RF [s]	XGB [s]
**i9-13900K**	445.90 ± 105.72	0.51 ± 0.01	1.17 ± 0.02
**RTX 2080**	14.10 ± 0.09 (32x)	0.77 ± 0.00 (0.66x)	0.98 ± 0.00 (1.19x)
**Tesla A16**	40.80 ± 0.00 (11x)	1.07 ± 0.00 (0.48x)	1.43 ± 0.00 (0.82x)
**RTX 4090**	3.44 ± 0.00 (130x)	0.76 ± 0.00 (0.67x)	0.84 ± 0.00 (1.39x)

**Table 2:** Comparison between classification times of our work with the state of the art.

	K-Means + SAM + SVM [16]	SVM (This Work)	XGB PU [33]	XGB GH13 [33]	XGB GH18 [33]	XGB (This Work)
**Time [s]**	~1	3.44	6.67	31.05	347.30	0.84
**# pixels**	From 300 to 1700	2500	207,400	664,845	5,014,744	2500
**# channels**	116	116	103	144	48	116

## Data Availability

Data available upon request to the corresponding author.
